# *Rpv3–1* mediated resistance to grapevine downy mildew is associated with specific host transcriptional responses and the accumulation of stilbenes

**DOI:** 10.1186/s12870-019-1935-3

**Published:** 2019-08-06

**Authors:** Birgit Eisenmann, Stefan Czemmel, Tobias Ziegler, Günther Buchholz, Andreas Kortekamp, Oliver Trapp, Thomas Rausch, Ian Dry, Jochen Bogs

**Affiliations:** 1 State Education and Research Center of Viticulture, Horticulture and Rural Development, Neustadt/Weinstr, Germany; 2 Centre for Organismal Studies Heidelberg, University of Heidelberg, Heidelberg, Germany; 3 Quantitative Biology Center (QBiC), University of Tübingen, Tübingen, Germany; 4 RLP AgroScience GmbH, AlPlanta - Institute for Plant Research, Neustadt/Weinstr, Germany; 5 Julius Kühn-Institute, Federal Research Centre of Cultivated Plants, Institute for Grapevine Breeding, Siebeldingen, Germany; 6 CSIRO Agriculture & Food, Urrbrae, SA 5064 Australia; 7 Technische Hochschule Bingen, 55411 Bingen am Rhein, Germany

**Keywords:** Disease resistance, Downy mildew, Grapevine, *Plasmopara viticola*, Stilbenes, *Vitis vinifera*, Metabolomics, *Rpv3*

## Abstract

**Background:**

European grapevine cultivars (*Vitis vinifera spp.) are* highly susceptible to the downy mildew pathogen *Plasmopara viticola*. Breeding of resistant *V. vinifera* cultivars is a promising strategy to reduce the impact of disease management. Most cultivars that have been bred for resistance to downy mildew, rely on resistance mediated by the *Rpv3* (*Resistance to* *P.* *viticola*) locus. However, despite the extensive use of this locus, little is known about the mechanism of *Rpv3*-mediated resistance.

**Results:**

In this study, *Rpv3*-mediated defense responses were investigated in *Rpv3*^+^ and *Rpv3ˉ* grapevine cultivars following inoculation with two distinct *P. viticola* isolates *avrRpv3*^+^ and *avrRpv3ˉ*, with the latter being able to overcome *Rpv3* resistance. Based on comparative microscopic, metabolomic and transcriptomic analyses, our results show that the *Rpv3–1*-mediated resistance is associated with a defense mechanism that triggers synthesis of fungi-toxic stilbenes and programmed cell death (PCD), resulting in reduced but not suppressed pathogen growth and development. Functional annotation of the encoded protein sequence of genes significantly upregulated during the *Rpv3–1*-mediated defense response revealed putative roles in pathogen recognition, signal transduction and defense responses.

**Conclusion:**

This study used histochemical, transcriptomic and metabolomic analyses of *Rpv3*^+^ and susceptible cultivars inoculated with avirulent and virulent *P. viticola* isolates to investigate mechanism underlying the *Rpv3–1*-mediated resistance response. We demonstrated a strong correlation between the expressions of stilbene biosynthesis related genes, the accumulation of fungi-toxic stilbenes, pathogen growth inhibition and PCD.

**Electronic supplementary material:**

The online version of this article (10.1186/s12870-019-1935-3) contains supplementary material, which is available to authorized users.

## Background

The biotrophic pathogen *Plasmopara viticola* (Berk. & M.A. Curtis) Berl. & de Toni causes grapevine downy mildew, one of the most prevalent grapevine diseases worldwide, leading to significant reductions in berry yield and quality [1]. Due to the lack of genetic resistance of *Vitis vinifera* species to downy mildew infection, wine production is heavily dependent on the use of fungicides to control this disease. To reduce the dependence of viticulture on chemical inputs, and thereby reduce the ecological and economic burden of wine production, a number of breeding programs have introgressed resistance loci from wild North American and Asian *Vitis* species into *V. vinifera* resulting in new downy mildew resistant grapevine cultivars [2, 3]. To date, 27 quantitative trait loci (QTL) conferring resistance to downy mildew have been identified within wild *Vitis* species [3–8]. However, to date, only the *Rpv1* resistance gene from *Muscadinia rotundifolia* has been cloned and functionally characterized. *Rpv1* is a NB-LRR receptor, involved in pathogen recognition and signal transduction during the initiation of plant defense [9]. Although 27 QTL regions associated with resistance against downy mildew are known, most downy mildew resistant cultivars grown in Europe rely on a single major resistance locus designated *Rpv3* (*Resistance to* *P.* *viticola*). The *Rpv3*-locus was first identified in *V. vinifera* cv. ‘Regent’ and described in more detail in *V. vinifera* cv. ‘Bianca’ [10–13]. Other new cultivars with *Rpv10* or *Rpv12*-mediated downy mildew resistance have been generated but are cultivated to a much lower extent. Further characterization of the previously identified *Rpv3* locus revealed allelic forms of this locus that all mediate resistance to downy mildew, referred to as *Rpv3–1, Rpv3–2* and *Rpv3–3* [13–15]. The *Rpv3*-mediated resistance is associated with the occurrence of necrotic lesions 48 to 72 h post inoculation (hpi), limited mycelial growth and a reduced number of new sporangiophores and sporangia [10, 12, 13, 16]. The cultivar ‘Regent’ (*Rpv3–1*) is a success story of resistance breeding and is one of the most cultivated downy mildew resistant varieties in Europe [17, 18]. However, despite the widespread use of the *Rpv3* resistance locus, detailed knowledge of the underlying mechanism of *Rpv3*-mediated resistance remains mostly unknown. Understanding the mechanism of resistance mediated by different resistance loci is essential for modern breeding strategies, as the combination of different resistance mechanisms in new grapevine cultivars could reduce the likelihood of breakdown of resistance by the pathogen [19]. Indeed, several studies have shown that *P. viticola* isolates have arisen in Europe that are able to overcome resistance mediated by the *Rpv3* locus [20–22]. In order to establish a successful colonization of grapevine leaves or berries *P. viticola* must suppress host plant defense mechanisms. It was demonstrated for different oomycetes that this suppression was achieved by the secretion of effector proteins [23] and a general model of plant defense was proposed by Dangl and Jones [24]. The detection of specific pathogen associated molecular patterns (PAMPs) by host pathogen recognition receptors (PRRs) leads to PAMP-triggered immunity (PTI) which is able to prevent non-adapted pathogens from successfully colonizing the plant and causing disease. However, host-adapted pathogens secrete effectors, which suppress PTI, leading to a compatible plant-pathogen interaction and host susceptibility (virulent pathogen isolates). During an incompatible plant-pathogen interaction, caused by avirulent pathogen isolates, these effectors are directly or indirectly recognized by specific resistance proteins with nucleotide-binding domains and leucine rich repeats (NB-LRR) resulting in a transcriptional activation of a variety of defense genes and a resistance of the plant to the pathogen (ETI; effector-triggered immunity) [25, 26]. Successful pathogen recognition leads to activation of signal transduction pathways involving MAP kinases and WRKY transcription factors, which in turn trigger primary immune responses such as accumulation of pathogenesis related (PR) proteins, reactive oxygen species (ROS) or phytoalexins, resulting in a hypersensitive response (HR) that prevents pathogen growth and development [27]. It has been demonstrated for different model organisms that a localized HR at the infection site is a common defense mechanisms observed during ETI [28, 29]. A clear distinction of the mechanisms underlying PTI and ETI cannot be made for all plant-pathogen interactions and some studies indicate overlaps of the defense response elicited by PTI and ETI [29]. For example, in *Arabidopsis thaliana*, the proteins involved in glucosinolate metabolism AtPEN2 and AtPEN3 are crucial to PTI and ETI [30–32]. It was also shown that degradation products of indole-glucosinolates, whose synthesis is mostly restricted to the order of Brassicacles, were involved in ETI-mediated HR [32]. However, it remains unclear if other bioactive secondary metabolites, could play a comparable role in plant defense in other plant species. For example, it has been proposed that stilbenes, which are secondary metabolites in grapevine, may play a similar role in grapevine defense [33]. The stilbene *trans*-resveratrol is the basic precursor from which all stilbenes found in grapevine are derived and is thus one of the most important stilbenes produced during plant defense [34, 35]. Various modifications of resveratrol result in the generation of bioactive derivatives including *ε-*viniferins (via oxidative dimerization) or *trans*-pterostilbene (via methylation). Previous studies have demonstrated the fungi-toxic effects of these stilbenes on *P. viticola* sporangia and zoospores [36–38]. In contrast, the glycosylated form of resveratrol, *trans*-piceid, was found to have only a very limited fungi-toxic effect on *P. viticola* sporangia or zoospores [37]. The induction of stilbene synthesis by various biotic and abiotic stresses such as inoculation with *Botrytis cinerea* or *P. viticola* or UV-C irradiation was observed in several grapevine varieties [39–43]. Furthermore, a number of previous studies have implicated a role for stilbene biosynthesis in resistance conferred by major *R* loci originating from wild North American and Asian grapevine species. For example, microarray analysis of the downy mildew resistant species *Vitis riparia* cv. Gloire de Montpellier revealed a multitude of *VvSTS* genes to be much more highly induced 12–24 hpi than in comparison to a susceptible *V. vinifera* cultivar [44]. Boso et al. [45] also observed much higher levels of stilbenes in *V. riparia* cv Gloire de Montpellier after downy mildew infection compared to *V. vinifera*. Correlations between resistance against *P. viticola* and high levels of *ε*-viniferin and *trans*-pterostilbene were also demonstrated for *Muscadinia rotundifolia* genotypes and an *Rpv10*-locus containing cultivar [46, 47]. Despite these previous publications implicating a role for stilbene biosynthesis in *R*-loci mediated resistance, not much is known about their role in *Rpv3–1*-mediated defense. In this study we have employed a novel approach to investigate this question by comparing downy mildew-induced stilbene biosynthesis, not only between different *Rpv3*^+^ and *Rpv3ˉ* grapevine genotypes, but also in response to inoculation with *P. viticola* isolates that are either virulent or avirulent on *Rpv3* genotypes. Our unique approach provides evidence that the *Rpv3*-mediated defense response involves the induction of the biosynthesis of fungi-toxic stilbenes, resulting in reduced, but not completely suppressed, pathogen growth and development.

## Results

### The *Plasmopara viticola* isolate *avrRpv3ˉ* overcomes *Rpv3*-mediated grapevine resistance

**Fig. 1:**
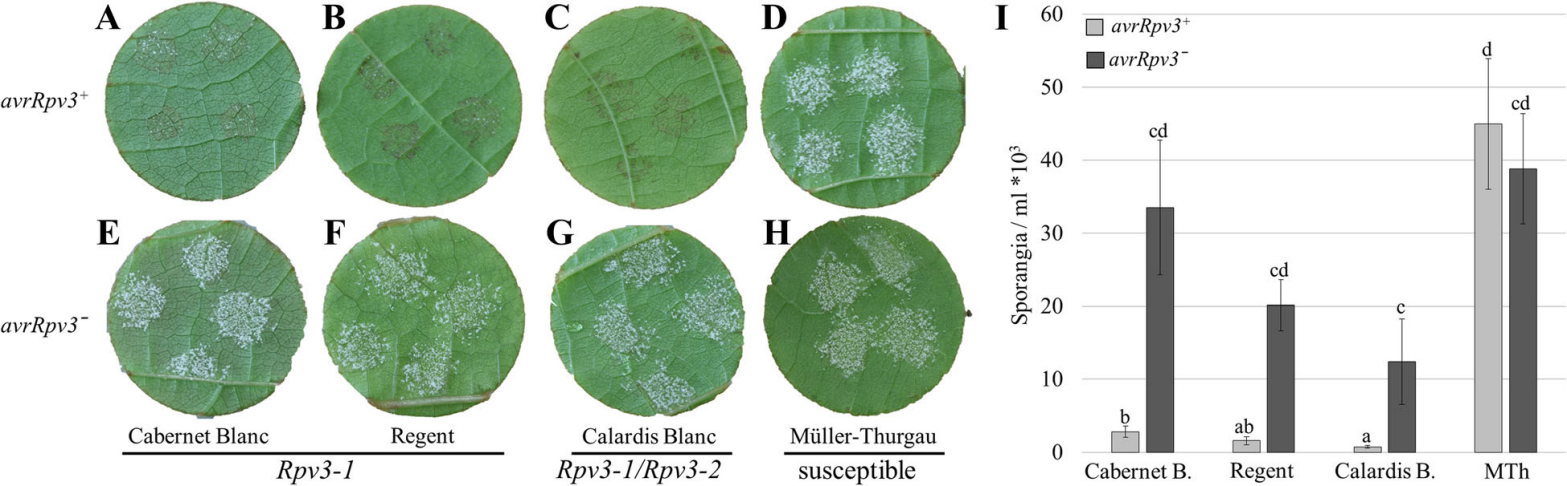
Growth and sporulation of virulent and avirulent *Plasmopara viticola* isolates on susceptible and *Rpv3* cultivars. Leaf discs of *Rpv3–1* cultivars (**a**, **e**) ‘Cabernet blanc’ and (**b**, **f**) ‘Regent’, (**c**, **g**) the *Rpv3–1/Rpv3–2* cultivar ‘Calardis blanc’ and (**d**, **h**) the susceptible cultivar ‘Müller-Thurgau’ were inoculated with the avirulent (*avrRpv3*^*+*^) (top) and virulent (*avrRpv3ˉ*) (bottom) *P. viticola* isolates. Pictures of representative leaf discs were taken at 6 days post inoculation (dpi). (I) Quantitative evaluation of sporulation of *P. viticola* isolates on leaf discs. Sporangia were counted 6 dpi. Bars represent the average of three independent experiments. Error bars show standard deviation. ANOVA was used to determine the effects of cultivar and treatment (the two isolates) on the amount of sporangia per ml*10^3^ and then means were compared by Tukey’s HSD (Honestly Significant Difference) test. For ANOVA and Tukey testing, sporangia count data was transformed to log values to fulfill criteria of normal distribution. Means with different letters (a, b, c, d) are significantly different (*P* < 0.05)

Downy mildew resistant grapevine cultivars containing the *Rpv3*-locus (‘Regent’ and ‘Cabernet Blanc’ - *Rpv3–1* and ‘Calardis Blanc’ - *Rpv3–1 & 3–2*) and the susceptible cultivar ‘Müller-Thurgau’ were inoculated with *P. viticola* isolates *avrRpv3*^*+*^ and *avrRpv3ˉ* to evaluate differences in host resistance against the two pathogen isolates. Resistance was assessed by observing the number of sporangia produced 6 days post inoculation (dpi) and the formation of necrotic lesions. After inoculation with the *avrRpv3*^*+*^ isolate, the number of sporangia produced on *Rpv3*^*+*^ cultivars was significantly lower (94–98% reduction) than that observed on the susceptible (*Rpv3ˉ*) cultivar (Fig. 1a-d, i). In contrast to the susceptible cultivar, necrotic areas were observed on the leaf discs of the *Rpv3*^*+*^ genotypes inoculated with the *avrRpv3*^*+*^ isolate (Fig. 1a-d). No necrotic spots were observed on leaf discs of any genotypes following inoculation with the *avrRpv3ˉ* isolate (Fig. 1e-h). The amount of *avrRpv3ˉ* sporangia was significantly higher in all *Rpv3* cultivars compared to the amount quantified after inoculation with the *avrRpv3*^*+*^ isolate, showing that the *avrRpv3ˉ* isolate is able to overcome *Rpv3*-mediated resistance. While, there was no statistically significant difference in the number of sporangia produced by the *avrRpv3ˉ* isolate across the different genotypes, the results strongly suggest a reduced susceptibility in ‘Calardis Blanc’ which contains both *Rpv3-1 & 3-2* compared to *Rpv3-1* only cultivars. For further studies ‘Regent’ was chosen as the representative *Rpv3–1* genotype (hereafter designated the *Rpv3–1* cultivar).

### *Rpv3–1*-mediated defense responses to avirulent and virulent *P. viticola* isolates

**Fig. 2:**
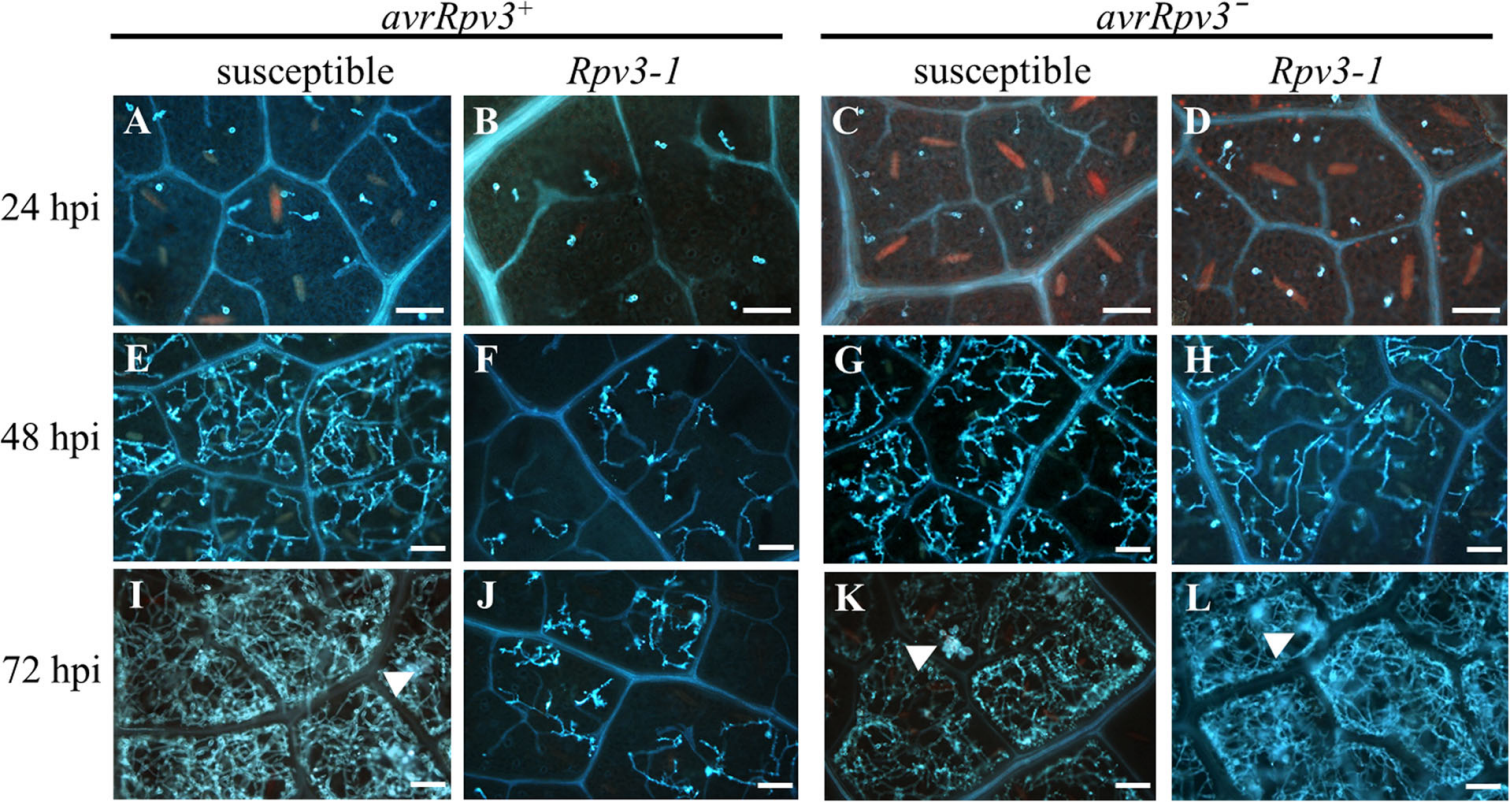
Comparison of *Plasmopara viticola* development in leaves of a susceptible and *Rpv3–1* cultivar. Time course of *P. viticola* development was evaluated using UV epifluorescence after aniline blue staining at 24 hpi (top), 48 hpi (middle) and 72hpi (bottom). Development of the avirulent (*avrRpv3*^*+*^) *P. viticola* isolate on leaf discs of the susceptible cultivar (**a**, **e**, **i**) and on *Rpv3–1* locus containing cultivar (**b**, **f**, **j**) and development of virulent (*avrRpv3ˉ*) *P. viticola* isolate on leaf discs of the susceptible grapevine cultivar (**c**, **g**, **k**) and on *Rpv3–1* locus containing cultivar (**d**, **h**, **l**) are shown. Arrows indicate sporangiophores. Images are representative of three biological replicates. Scale bars correspond to 100 μm

For histochemical analysis of *Rpv3–1-*mediated host resistance and pathogen development, the *Rpv3–1* and susceptible cultivars were inoculated with the *avrRpv3*^*+*^ and *avrRpv3*^*¯*^ *P. viticola* isolates and samples collected 24, 48 and 72 h post inoculation (hpi). Leaf discs were stained with aniline blue to monitor the time course of *P. viticola* development (Fig. 2). No differences were observed in the early colonization phase between cultivars or between *P. viticola* isolates. By microscopically observations comparable zoospore attachment to stomata, germ tube development, formation of primary hyphae and development of haustoria were observed in all treatments at 24 hpi (Fig. 2a-d, Additional file 1). At 48 hpi, mycelial growth of the *avrRpv3*^*+*^ isolate was markedly impaired in the *Rpv3–1* cultivar, compared to the susceptible cultivar (Fig. 2e, f). However, growth of the *avrRpv3ˉ* isolate was similar within the intercellular spaces of the susceptible and *Rpv3–1* cultivars (Fig. 2g, h). At 72 hpi, the spongy mesophyll of the susceptible cultivar was entirely colonized by the mycelium and sporangiophores had been produced by both isolates, signifying a successful pathogen life cycle (Fig. 2i, k). In contrast, only weak mycelial growth and no sporangiophore formation was observed after 72 hpi for the *avrRpv3*^*+*^ isolate on the *Rpv3–1* cultivar (Fig. 2j), whereas growth and sporulation of the *avrRpv3ˉ* isolate on the *Rpv3–1* cultivar was similar to that observed on the susceptible cultivar (Fig. 2k, l).

**Fig. 3:**
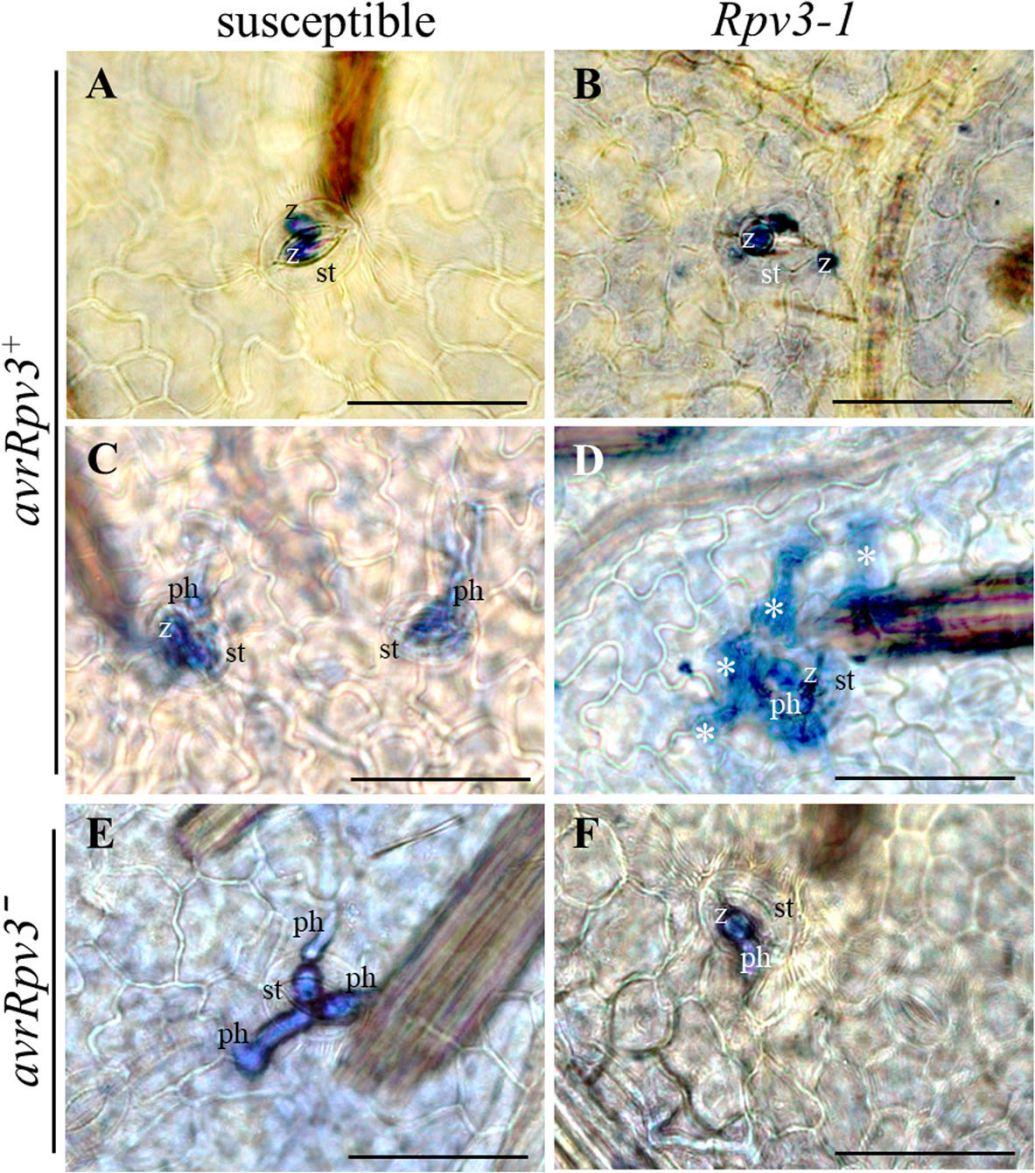
Induction of programmed cell death at the *Plasmopara viticola* infection site. Leaf discs of a susceptible cultivar (**a**, **c**, **e**) and an *Rpv3–1* cultivar (**b**, **d**, **f**) were inoculated with the avirulent (*avrRpv3*^*+*^) *P. viticola* isolate and samples were taken at 24 hpi (**a**, **b**) and 32 hpi (**c**, **d**). Leaf discs were inoculated with the virulent (*avrRpv3ˉ*) *P. viticola* isolate and samples were collected at 48 hpi (**e**, **f**). Abbreviations: st, stomata; z, encysted zoospore; ph, primary hyphae; asterisks indicate trypan blue-stained cells undergoing PCD in response to *P. viticola* infection. Images are representative of three biological replicates. Scale bars correspond to 50 μm

In addition to examine pathogen development, the occurrence of host programed cell death (PCD) at infection sites was also examined using trypan blue staining (Fig. 3). Even though this staining method was optimized for visualization of PCD, some *P. viticola* structures were co-stained allowing the identification of infected stomata. At 24 hpi with the *avrRpv3*^*+*^ isolate, encysted zoospores were present at stomata of both cultivars, but no trypan blue-stained cells were visible, indicating that PCD had not been initiated (Fig. 3a, b). At 32 hpi, PCD was clearly visible in mesophyll cells below the infected stomata in the *Rpv3–1* cultivar inoculated with the *avrRpv3*^*+*^ isolate, but no PCD was observed in the susceptible cultivar (Fig. 3c, d). In addition, no PCD was observed in any leaf disc of the susceptible or *Rpv3–1* cultivars up to 48 hpi with the *avrRpv3ˉ* isolate (Fig. 3e, f). This histochemical analysis indicate that the *Rpv3–1-mediated* defense results in restriction of pathogen growth and development that initiates later than 24 hpi and is effective before 48 hpi with PCD at 32 hpi.

### Expression of stilbene biosynthesis genes correlates with stilbene accumulation after *Plasmopara viticola* infection in *Rpv3–1* cultivar

**Fig. 4:**
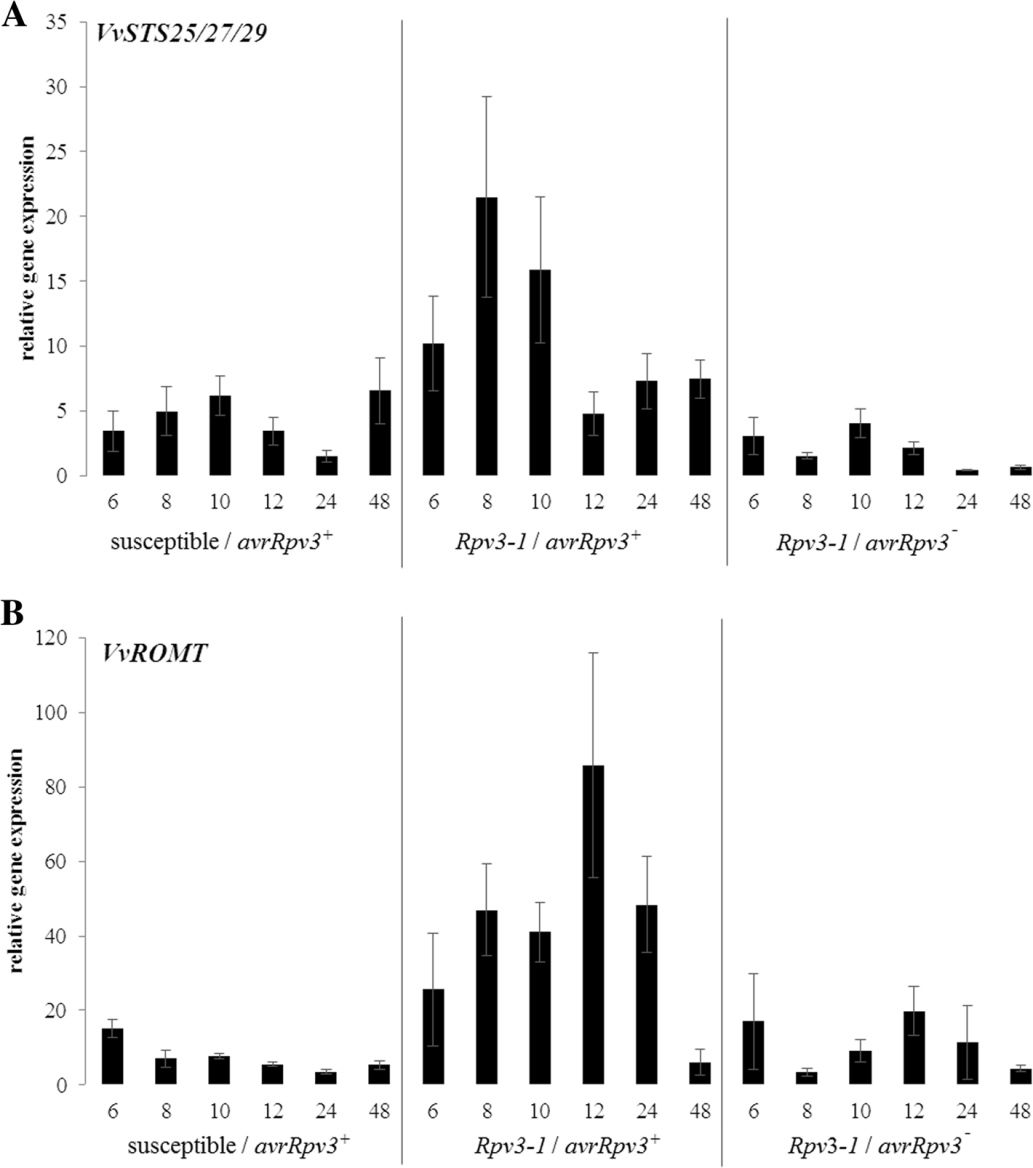
Relative gene expression in leaf discs of susceptible and *Rpv3–1* cultivars inoculated with *Plasmopara viticola*. Time course of gene expression was determined by qPCR and normalized to grapevine housekeeping genes and mock treatment. x axis shows hours post infection (hpi). Genes involved in stilbene biosynthesis (**a**) *VvSTS25/27/29* and (**b**) *VvROMT* were evaluated. Bars represent the average of two independent measurements of triplicates of five pooled biological replicates. Error bars show standard deviation

To gain insights into the possible role of stilbene pathway genes in *Rpv3–1*-mediated resistance, the expression profiles of a number of different genes involved in stilbene biosynthesis were studied by qPCR in the susceptible and *Rpv3–1* cultivars after inoculation with the two *P. viticola* isolates (*avrRpv3*^*+*^ & *avrRpv3ˉ*) or water. Gene expression was calculated relative to the water controls and normalized against grapevine housekeeping genes. One primer set (*VvSTS25/27/29)* was used to quantify the combined transcript levels of *VvSTS25*, *VvSTS27* and *VvSTS29,* encoding for putative stilbene synthases, which have been shown previously to be highly responsive to biotic and abiotic stress [48]*.* Additionally the transcript level of *VvROMT*, which encodes a resveratrol *O*-methyltransferase catalyzing *trans*-pterostilbene biosynthesis [38, 49], was also analyzed. Transcription of *VvSTS* and *VvROMT* genes were found to be strongly up-regulated, within the first 24 hpi, in grapevine tissues undergoing a resistance response (*Rpv3–1*/*avrRpv3*^*+*^) when compared to tissues undergoing susceptible interactions (i.e. susceptible/*avrRpv3*^*+*^ and *Rpv3–1*/*avrRpv3¯*) (Fig. 4). The successful induction of resistance in the *Rpv3–1* cultivar inoculated with the *avrRpv3*^*+*^ isolate was associated with a peak of *VvSTS* and *VvROMT* transcription at 8 and 12 hpi, respectively. In contrast, the expression of these genes in the *Rpv3–1* cultivar inoculated with virulent *avrRpv3ˉ* isolate or in the susceptible cultivar was relatively constant and lower across the entire infection time course. For example, a clear induction was measured for *VvSTS25/27/29* (17 fold) and *VvROMT* (14 fold) in the *Rpv3–1* cultivar at 8 hpi inoculated with *avrRpv3*^*+*^ compared to leaf discs inoculated with *avrRpv3ˉ* (Fig. 4)*.* Having demonstrated a significant induction of stilbene biosynthesis pathway genes associated with grapevine leaf tissue undergoing an *Rpv3–1*-mediated defense response, the next step was to investigate whether this translated into significant differences in the levels and diversity of stilbene compounds within the tissues undergoing avirulent and virulent interactions.

### Activation of *Rpv3–1*-mediated defense is associated with induction of stilbene biosynthesis

**Fig. 5:**
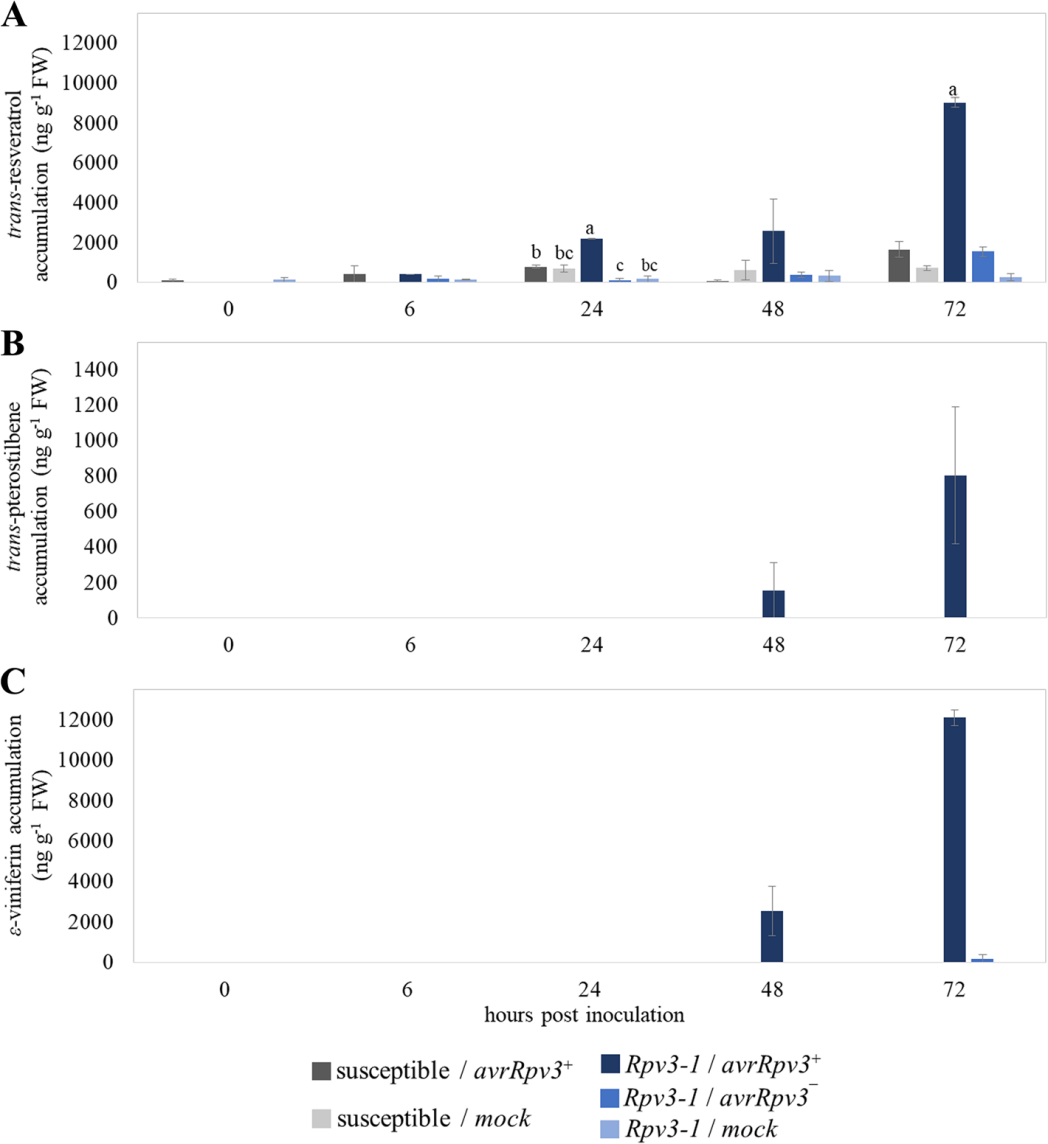
Accumulation of stilbenes in susceptible and *Rpv3–1* cultivars in response to *Plasmopara viticola* inoculation. **a** *trans*-resveratrol, **b** *trans*-pterostilbene, and **c** *ε*-viniferin were measured in leaf discs after inoculation with *P. viticola* isolates (*avrRpv3*^*+*^ or *avrRpv3¯*) or water control (H_2_O). Samples were collected 0, 6, 24, 48 and 72 hpi. Bars represent the average of two independent measurements of five pooled biological replicates. Error bars show standard deviation. ANOVA was used to determine the effects of cultivar and treatment (the two isolates) on the stilbene amount and then means were compared by Tukey’s HSD test. Statistical analysis is related to significance of all samples at the same time point, different letters (**a**, **b**, **c**) are significantly different (*P* < 0.05)

The level of the four stilbene compounds *trans*-resveratrol, *ε*-viniferin, *trans*-pterostilbene and *trans*-piceid was determined by HPLC over a 72 h period. Commencing at 24 hpi the successful induction of *Rpv3–1*-mediated defense response against the *avrRpv3*^*+*^ isolate is associated with a significant higher level of *trans*-resveratrol, when compared to the infection of the *Rpv3–1* cultivar with *avrRpv3ˉ* isolate and the susceptible cultivar with *avrRpv3*^*+*^ or water controls (Fig. 5). The accumulation of *trans*-resveratrol, the precursor molecule for stilbenes like *trans*-piceid, *ε*-viniferin or *trans*-pterostilbene was about six fold induced in a successful pathogen recognition and plant defense (*Rpv3–1*/*avrRpv3*^*+*^), when comparing 6 and 24 hpi (Fig. 5a). This resulted in a significant higher amount of resveratrol at 24 hpi (~ 2.180 ng g^− 1^ FW). In contrast, the level of *trans*-resveratrol detected in corresponding *Rpv3–1* samples inoculated with *avrRpv3ˉ* or water at 6 and 24 hpi did not change markedly (Fig. 5a) The amount of *trans*-resveratrol in *Rpv3–1*/*avrRpv3*^*+*^ samples further increased to 9.000 ng g^− 1^ FW at 72 hpi, resulting in a significantly higher amount of resveratrol during successful defense compared to the inoculated susceptible cultivar (~ 1.600 ng g^− 1^ FW) or *Rpv3–1* cultivar inoculated with *avrRpv3ˉ* (~ 1.500 ng g^− 1^ FW), respectively (Fig. 5a). *Trans*-resveratrol was also detected in corresponding water controls showing a significant lower amount compared to corresponding time points of the *Rpv3–1*/*avrRpv3*^*+*^ treatment. In contrast, no significant differences were found when comparing the corresponding time points of water controls, with *Rpv3–1*/*avrRpv3ˉ* treated samples or susceptible samples (Fig. 5a). Of particular interest was the finding that the two most fungi-toxic stilbenes, *ε*-viniferin and *trans*-pterostilbene only accumulated during a successful defense at 48 and 72 hpi, resulting in approximately 800 ng g^− 1^ FW *trans*-pterostilbene and 12000 ng g^− 1^ FW *ε*-viniferin at 72 hpi (Fig. 5b, c). A small amount of ε-viniferin (~ 180 ng g^− 1^ FW) was also detected in samples inoculated with the *avrRpv3ˉ* isolate at 72 hpi, but this was approximately 70 fold lower than the amount found during in leaf tissues undergoing a successful defense response (*Rpv3–1*/*avrRpv3*^*+*^). The stilbene *trans*-piceid is the glycosylated form of *trans*-resveratrol and is considered as transport and storage form of stilbenes without fungi-toxic effects on *P. viticola* [37]. *Trans*-piceid was found in all samples, independent of time point, treatment and cultivar indicating that the concentration of this stilbene might not be important for a successful defense against *P. viticola* (Additional file 2). These results indicate that the significantly higher accumulation of *trans*-resveratrol and the specific biosynthesis of the fungi-toxic stilbenes *ε*-viniferin and *trans*-pterostilbene in *Rpv3–1* cultivar are associated with the successful activation of the *Rpv3–1*-mediated defense mechanism in grapevine leaf tissues against *P. viticola*.

### Transcriptomic analysis of differentially expressed genes in response to *Plasmopara viticola* infection

**Fig. 6:**
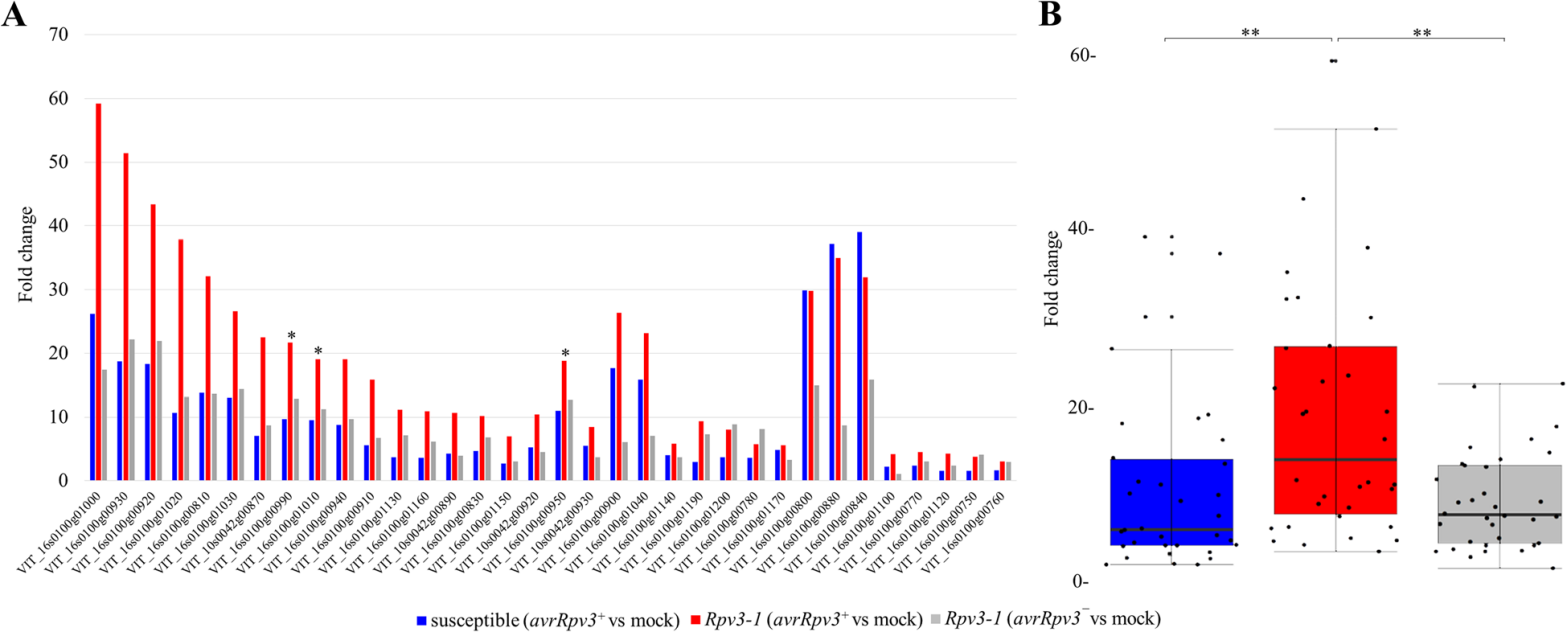
Global expression analysis of the stilbene synthase gene family in response to *Plasmopara viticola* infection. **a** Fold change (infected vs mock treatment) of 34 differentially expressed grapevine stilbene synthase genes in *Rpv3–1/avrRpv3*^*+*^, susceptible*/avrRpv3*^*+*^ and *Rpv3–1/avrRpv3ˉ* samples at 6 hpi as determined by RNA-Seq analysis. Asterisks mark stilbene synthase genes evaluated by qPCR: *VvSTS25* (VIT_16s0100g00950), *VvSTS27* (VIT_16s0100g00990) and *VvSTS29* (VIT_16s0100g01010). **b** Box plots showing the fold change of all 34 differentially expressed stilbene synthase genes. The median is indicated by the horizontal line in the box. Whiskers represent 95% confidence intervals. Asterisks show significant differences (*P* < 0.01)

**Fig. 7:**
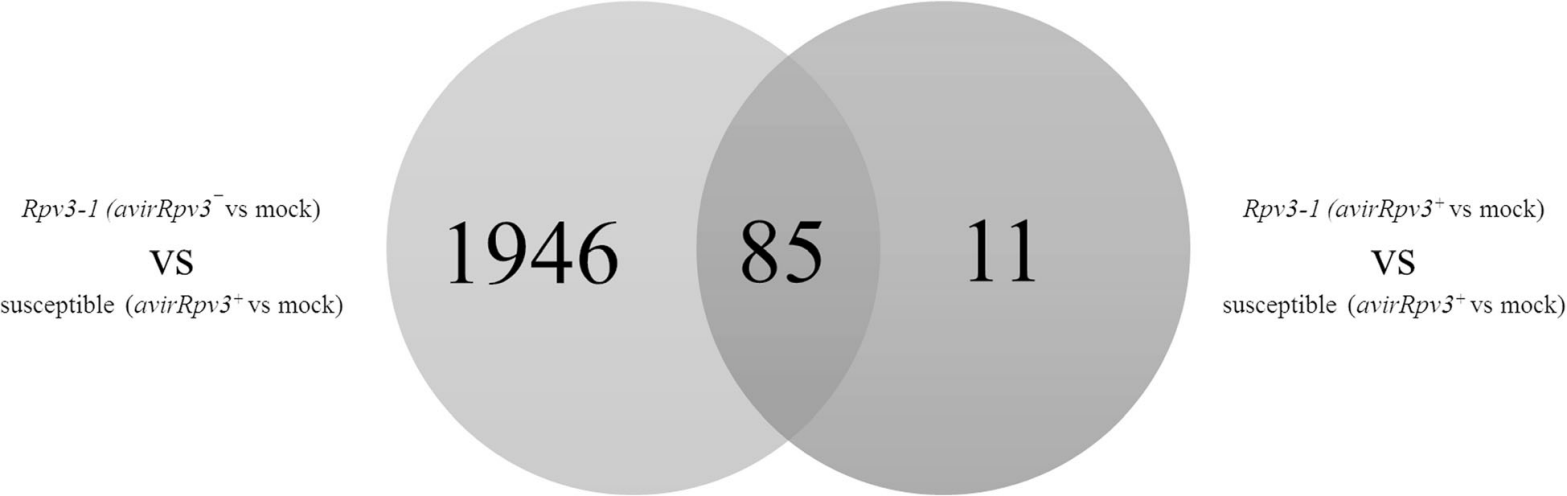
Venn diagram of differentially expressed genes of *Vitis vinifera* cultivars in response to *Plasmopara viticola*. On top, the Venn diagram shows the genes differentially expressed (DE) in the interaction term *Rpv3–1/avrRpv3*^*+*^ versus mock compared to susceptible/*avrRpv3*^*+*^ versus mock and below the Venn diagram shows genes DE in the interaction term *Rpv3–1/avrRpv3¯* versus mock compared to susceptible/*avrRpv3*^*+*^ versus mock. In total, 2042 genes were differentially expressed

Gene expression analysis of selected *VvSTS* genes showed the highest induction during a successful *Rpv3–1*-mediated defense between 6 hpi and 8 hpi (Fig. 4). In order to try and identify other genes involved in the *Rpv3–1*-mediated defense response, a non-targeted approach was employed using RNA-Seq analysis to identify differentially expressed genes in susceptible and *Rpv3–1* cultivars 6 hpi with *P. viticola* isolates *avrRpv3*^*+*^, *avrRpv3ˉ* or water. RNA-Seq data was first analyzed using a simple pairwise comparison method to identify genes that exhibit a significant differential expression in response to *P. viticola* infection when compared to mock-treated samples of each cultivar. Statistical analysis identified 2612 genes that were differentially expressed with respect to the mock control in at least one pairwise comparison, based on a false discovery rate (FDR) < 10% (multiple adjusted *p* value *P* < 0.1) and a minimum log fold-change (logFC) of 1 (Additional file 3). RNA-Seq results were validated by qPCR analysis (Additional file 4) for the *P. viticola* induced genes *VvPR10.1*, *VvPR5*, *VvROMT* and *VvSTS1.* The results demonstrated a strong significant correlation (r = 0.95–0.99) between qPCR and RNA-Seq data (Additional file 4). Among those 2612 differentially expressed (DE) genes, 34 were found to encode stilbene synthase proteins (Fig. 6a). Of these 34 *VvSTS* genes, 26 were identified as being more highly induced in leaf tissues undergoing a successful *Rpv3–1*-mediated defense (*Rpv3–1*/*avrRpv3*^*+*^) and include the *VvSTS* genes shown to be up-regulated by qPCR in Fig. 4. Even though most of the individual *VvSTS* genes are statistically significantly induced by infection in all samples, a significant difference was found when comparing the gene expression of all 34 *VvSTS* genes (Fig. 6b). This revealed that the total *VvSTS* expression in *Rpv3–1*/*avrRpv3*^*+*^ was significantly higher compared to susceptible and *Rpv3–1*/*avrRpv3ˉ* samples (Fig. 6b). The pairwise comparison method applied above is only able to identify DE genes between inoculated and mock samples of either the susceptible or the *Rpv3–1* cultivar but is not suitable to identify genes whose response to infection is statistically significant different between the cultivars. In order to identify candidates differentially expressed during a successful *Rpv3–1*-mediated defense response, differential expression analysis was performed using linear modelling including interaction terms. These interaction terms made it possible to identify DE genes between the *Rpv3–1*/*avrRpv3*^*+*^ samples and the susceptible/*avrRpv3*^*+*^ samples (= successful defense) as well as genes that are differentially expressed between the *Rpv3–1*/*avrRpv3ˉ* samples and the susceptible/*avrRpv3*^*+*^ samples (= unsuccessful defense). This analysis revealed a total of 2042 DE genes and a Venn diagram was drawn to show the overlap between these two comparisons (Fig. 7, Additional file 5). A total of 85 genes were found to be common between the successful and the unsuccessful defense responses indicating that these genes were differentially expressed in *Rpv3–1* samples independent from *P. viticola* isolates. The analysis indicates that 11 genes specifically expressed in samples undergoing a successful pathogen recognition and defense (*Rpv3–1*/*avrRpv3*^*+*^), whereas 1946 genes were found to be differentially regulated in *Rpv3–1* samples inoculated with the virulent isolate (Fig. 7). This group of 11 genes are of special interest for further studies as they are differentially expressed only during the early stages (6 hpi) of a successful *Rpv3–1*-mediated defense response (Table 1). Functional annotation of the encoded protein sequences of the 11 genes in this group showed them to have predicted putative functions as aspartyl proteases (VIT_04s0008g07150; VIT_04s0008g07250), peroxidase (VIT_12s0055g01000), metal-nicotianamine transporter (VIT_16s0098g01250), lipase (VIT_10s0003g02120) and chitinase (VIT_05s0062g01320), a MUTL protein homolog (VIT_04s0044g00170), a Zinc knuckle family protein (VIT_05s0020g00290), a Leucine Rich Repeat receptor-like kinase (VIT_12s0034g02570) and two unknown proteins (VIT_18s0001g07610; VIT_14s0060g02120) (Table 1).

**Table 1:** Differentially expressed (DE) genes in an *Rpv3–1* cultivar undergoing a successful defense. Samples were collected 6 hpi with the avirulent (*avrRpv3*^*+*^) or virulent (*avrRpv3ˉ*) *Plasmopara viticola* isolate and water treatment. These eleven DE genes were identified by analyzing the interaction terms of *Rpv3–1/avrRpv3*^*+*^ *(avrRpv3*^*+*^ vs *mock)* vs susceptible *(avrRpv3*^*+*^ vs *mock)* and *Rpv3–1/avrRpv3ˉ (avrRpv3ˉ* vs *mock)* vs susceptible *(avrRpv3*^*+*^ vs *mock)* samples and represent genes that are differentially expressed only in the avirulent interaction (*P* < 0.1)

Gene ID	Functional annotation	*Rpv3-1/avrRpv3*^+^ vs susceptible		*Rpv3-1/avrRpv3*¯ vs susceptible	
		logFC	adj. *P* value	logFC	adj. *P* value
VIT_04s0008g07150	Aspartyl protease	3390	0.098	1622	0.143
VIT_04s0008g07250	Aspartyl protease	2862	0.082	1417	0.102
VIT_12s0055g01000	Peroxidase	1666	0.095	–0.460	0.338
VIT_16s0098g01250	Metal-nicotianamine transporter YSL3	1600	0.082	0.311	0.435
VIT_10s0003g02120	Lipase GDSL	1570	0.082	–0.123	0.758
VIT_05s0062g01320	Chitinase	1069	0.095	0.566	0.103
VIT_14s0060g02120	Unknown	–0.937	0.095	–0.382	0.181
VIT_04s0044g00170	MUTL protein homolog 3 (MLH3)	–1,013	0.082	–0.491	0.105
VIT_05s0020g00290	Zinc knuckle family protein	–1090	0.098	–0.312	0.330
VIT_12s0034g02570	Leucine Rich Repeat receptor-like kinase	–1488	0.077	–0.528	0.154
VIT_18s0001g07610	Unknown	–1616	0.095	–0.714	0.155

## Discussion

### Histological evaluation of *Rpv3*-mediated resistance in response to virulent and avirulent *P. viticola* isolates

In this study, the mechanism of *Rpv3*-mediated resistance against *P. viticola* was evaluated by comparing the induction of defense responses of susceptible and *Rpv3* resistant grapevine cultivars after inoculation with avirulent (*avrRpv3*^*+*^) or virulent (*avrRpv3ˉ*) *P. viticola* isolates. The results revealed that *Rpv3-*mediated resistance relies on inducible responses specifically elicited by the avirulent (*avrRpv3*^*+*^) strain, resulting in necrotic lesions and reduced sporulation (Fig. 1), which has been previously described for other *P. viticola* isolates that are virulent and avirulent on *Rpv3* genotypes [16, 20]. Aniline blue staining revealed that zoospores from both isolates were able to encyst at stomata and developed primary hyphae in a comparable manner on both the *Rpv3–1* and the susceptible cultivars (Fig. 2, Additional file 1). These results are consistent with the previous findings of Kortekamp et al. [50] and indicate that *Rpv3–1-*mediated resistance relies on inducible responses presumably provoked by the first interaction of plant cells and pathogen hyphae rather than on constitutive defense mechanisms [44]. One of the most studied localized plant response upon pathogen recognition is PCD, which is visible as necrotic lesions at the infection site [51]. The presence of necrotic lesions within 2–10 days after *P. viticola* infection has been described for several resistant grapevine genotypes with different levels of resistance and it has been speculated whether these differences could be explained by differences in the speed of initiation of PCD which effectively denies the biotrophic oomycete pathogen of nutrition [12, 21, 42, 52, 53]. To our knowledge, this is the first study, presenting a detailed evaluation of the timing of occurrence of downy mildew-triggered PCD in a resistant grapevine genotype. The first differences between a successful and an unsuccessful *Rpv3–1*-mediated defense response were observed at 32 hpi with the induction cell death, which was followed by inhibition of mycelial growth in the *Rpv3–1* cultivar inoculated with the avirulent *P. viticola* isolate (Figs. 2-3). A clear difference in pathogen development was observed at 48 hpi, which resulted in marked reduction, but not complete suppression, of downy mildew sporulation all *Rpv3–1* cultivars examined (Fig. 1). As grapevines with different origins and *Rpv-*loci, display a wide range of resistance levels [54], time course studies of PCD progression across these host species could lead to a better understanding of differences in resistance mechanisms and importance of the temporal onset of PCD on *P. viticola* development in these genotypes. A number of different *P. viticola* isolates have previously been identified that were able to overcome *Rpv3-*mediated resistance, demonstrating that the durability conferred by a single resistance locus can be low [16, 20, 22, 55]. The emergence of resistance-breaking pathogens in resistant crops is a well described process during which pathogens can become virulent by evolution of their avirulence genes. As a consequence, resistance proteins are no longer able to recognize these altered avirulent proteins (effectors) [24]. Resistance-breaking isolates develop due to the selection pressure, exerted by plant resistance genes and have been observed in a multitude of crops such as potato and rice [56, 57]. The *avrRpv3ˉ P. viticola* isolate we describe is capable of breaking *Rpv3–1-*mediated resistance (Figs. 1, 2, 3) suggesting that mutated avirulence protein (*avrRpv3*) is not recognized by the corresponding *R* gene product of the *Rpv3–1-*locus. The amount of new sporangia produced by this virulent isolate was significantly higher in all *Rpv3* cultivars compared to the avirulent isolate. However, Regent (*Rpv3–1*) and Calardis Blanc (*Rpv3–1* & *3–2*) show differences in mean amount of sporangia when compared to Cabernet blanc (*Rpv3–1*), which could hint at an elevated level of resistance against the virulent isolate mediated by the presence of additional minor loci [10, 11]. However, it is clear that the *avrRpv3ˉ* isolate used in this study was still able to overcome the resistance mediated by *Rpv3–1* and *Rpv3–2,* suggesting that these two *R* loci may recognize the same *avr* effector. Further experiments with genotypes containing *Rpv3–2,* in the absence of *Rpv3–1,* are required to determine whether the *Rpv3–2*-mediated resistance is also compromised by this *avrRpv3ˉ* isolate. The combination (pyramiding) of different *R* loci is recognized as an important strategy to increase durability of resistance against plant pathogens [2, 58]. An understanding of the mechanisms underlying *R* gene mediated resistance and the recognized *avr* effectors will be crucial role in finding successful resistance loci combinations to guarantee a durable resistance against grapevine downy mildew.

### Stilbenes and their role in the *Rpv3–1*-mediated defense

The induction of secondary metabolites in response to biotic and abiotic stresses is a well-known defense reaction. In grapevine, stilbenes are a class of stress-induced secondary metabolites that are commonly involved in responses to various biotic and abiotic stresses [33, 36, 39, 42, 46]. Stilbene synthase (STS) represent the first committed enzyme step in the biosynthesis of stilbenes catalyzing the synthesis of resveratrol [43, 59, 60]. In grapevine, the *VvSTS* family consists of forty-eight putative *VvSTS* gene sequences with at least thirty-three full-length sequences encoding potentially functional proteins [43].

Using qPCR analysis it was possible to evaluate the expression level of different *VvSTS* genes within the first 48 hpi with downy mildew (Fig. 4). The qPCR analysis revealed that these genes were expressed on a comparable and relatively constant level in the compatible interactions (*Rpv3–1/avrRpv3*^*¯*^ & susceptible/*avrRpv3*^*+*^) over the 48 h infection period and were associated with the accumulation of *trans*-resveratrol and the non-toxic *trans*-piceid (Fig. 5). In contrast, *VvSTS* genes were highly induced within 6–8 hpi in the incompatible (*Rpv3–1/avrRpv3*^*+*^) interaction which resulted in a successful defense response (Fig. 4). This was further confirmed by RNA-Seq analysis of leaf tissue sampled at 6 hpi which confirmed an elevated level of transcription in a total of 34 *VvSTS* genes in the incompatible interaction (Fig. 6). However, interaction term analysis showed that the direction of regulation and strength is not statistically different between *Rpv3–1/avrRpv3*^*+*^ and *Rpv3–1/avrRpv3*^*−*^ samples when compared to susceptible plants which indicates the induction of *VvSTS* genes in general is not specific to a successful defense. Still, when looking at the overall fold changes across the whole set of *VvSTS* genes (Fig. 6b), it was demonstrated that during a successful defense (in *Rpv3–1/avrRpv3*^*+*^ samples) a network of *VvSTS* genes is upregulated even further than in susceptible samples. It can be speculated that this results in synthesizing a higher level of *trans*-resveratrol that provides the precursors for additional biosynthetic reactions leading to the production of the oligomeric stilbenes *ε*-viniferin and *trans*-pterostilbene. Gene expression analysis of the resveratrol O-methyltransferase (VIT_12s0028g01880), a gene that encodes a protein responsible for the biosynthesis of *trans*-pterostilbene from resveratrol [38] also revealed a higher level of expression in leaf tissues undergoing a successful *Rpv3–1*-mediated defense response (Fig. 4 for qPCR, Additional file 3 for RNA-Seq) compared to susceptible samples. Thus, the expression data of stilbene biosynthesis-related genes shows a strong correlation with the detectable levels of stilbene compounds (Fig. 5).

The toxicity of the different stilbenes on sporangia or zoospores of *P. viticola* has been previously investigated [36, 61]. These studies showed that *trans*-piceid had no toxicity and *trans*-resveratrol only low toxicity on *P. viticola* sporangia and zoospores. In contrast, *ε*-viniferin and *trans*-pterostilbene were found to have strong fungi-toxic effects on grapevine downy mildew. However, conclusive evidence of a direct role for stilbenes in reducing the susceptibility of certain grapevine genotypes to *P. viticola* infection is still lacking. It has previously been shown that whereas the stilbenes *trans*-resveratrol and *trans*-piceid may be induced in both susceptible and downy mildew-resistant cultivars, the fungi-toxic oligomeric forms (*ε*-viniferin and *trans*-pterostilbene) are found exclusively, or at much higher levels, in downy mildew-resistant cultivars [35, 42, 46, 47, 62]. Similarly, our results show that *ε*-viniferin and *trans*-pterostilbene were detected exclusively in leaf discs displaying a successful defense response against the avirulent *P. viticola* isolate (Fig. 4) strongly suggesting a role for these compounds in *Rpv3–1*-mediated defense. While *trans*-resveratrol has only low toxicity to *P. viticola*, it may have another role in *Rpv3–1*-mediated defense other than as a precursor of viniferin and *trans*-pterostilbene biosynthesis. Chang et al. [63] showed that the addition of exogenous *trans*-resveratrol inhibited the growth of *Vitis* cell suspension cultures and activated defense-related responses such as ROS formation and cell death. They postulated that *trans*-resveratrol could itself act as a signaling molecule initiating PCD. Interestingly, we observed the first cells undergoing PCD in *Rpv3–1* genotypes at 32 hpi (Fig. 3), not long after the appearance of elevated levels of *trans*-resveratrol (Fig. 5). Vezzulli et al. [14] recently demonstrated a correlation between *Rpv3–3* locus-mediated resistance against downy mildew and the induction of oligomeric stilbenes in a ‘Merzling’ x ‘Teroldego’ segregating population. They postulated that downy mildew resistance in this population was likely mediated by the combined action of the *Rpv3–3* locus and stilbene biosynthesis. The results presented here complement their findings by showing that induction of stilbene biosynthesis pathway genes and the accumulation of oligomeric fungi-toxic stilbenes are specifically upregulated following recognition of the *avrRpv3* effector by *Rpv3–1* and are likely to be an important component of *Rpv3*-mediated defense. Ultimately, conclusive proof of a role for stilbenes in *Rpv3*-mediated resistance can only be obtained by studying the downy mildew resistance of *Rpv3* genotypes in which the stilbene synthase gene family has been deleted or silenced which would be particularly challenging given the large number of *VvSTS* genes in the grapevine genome [43].

### Early specific transcriptomic responses of the *Rpv3*-mediated resistance mechanism

The first transcriptional defense responses of *Rpv3–1* cultivar ‘Regent’ have been reported between 6 and 8 hpi here and in other studies [64, 65]. In order to discover other transcriptional and biochemical pathways, in addition to the stilbene biosynthesis pathway that might be involved in *Rpv3–1*-mediated downy mildew resistance we also compared early (6 hpi) transcriptomic responses of leaf tissues undergoing compatible and incompatible interactions with *P. viticola*. Evaluation of *Rpv3–1*-mediated transcriptional responses by RNA-Seq analysis confirmed the induction of a large number of host genes in both interactions, although this occurs for a number of genes with greater intensity in the incompatible interaction [42, 64]. However, it is difficult to draw any conclusions from these results because of the influences of genomic background of the host plants on differences in gene expression cannot be excluded. Most transcriptional studies that set out to identify genes specifically involved in *R* gene-mediated resistance are based on comparisons of gene expression between a resistant genotype that contains the *R* gene and a susceptible genotype that doesn’t. However, the analysis in this case is complicated by differences in gene expression arising from the different genetic backgrounds of the host species. Therefore, our approach was to not only compare the transcriptional responses of susceptible and resistant genotypes, but also use an *Rpv3* resistance-breaking *P. viticola* isolate (*avrRpv3¯*) to compare transcriptomic response of the same *Rpv3–1* cultivar undergoing a successful and unsuccessful defense. In order to analyze the RNA-Seq data comprehensively, statistics was done in two parts. In a first statistical approach, RNA-Seq data was analyzed using pairwise comparisons between *P. viticola* and mock treated samples in order to identify DE genes in response to *P. viticola* infection irrespective of the genotypes. Secondly, a more sophisticated statistical approach using linear modeling including interaction terms was performed in order to compare the differential gene expression responses in the *Rpv3–1*/*avrRpv3*^*+*^ samples compared to susceptible/*avrRpv3*^*+*^ samples (= successful defense) and the differential gene expression responses in *Rpv3–1*/*avrRpv3ˉ* samples compared the susceptible/*avrRpv3*^*+*^ samples (= unsuccessful defense). In the first approach using simple pairwise comparisons a total of 2612 genes were DE with respect to the mock control in at least one pairwise comparison. Using the second more stringent statistical approach many genes were excluded whose DE is influenced by events unrelated to *Rpv3–1*-mediated defense mechanism. In total 2042 DE were found in this approach. Interestingly, only one of the previously identified 34 STS genes which showed different expression in the pairwise comparison (infected vs mock) was DE comparing the different genotypes (interaction term analyses). This is in line with results depicted in Fig. 4 showing a positive regulation for *STS* genes upon treatment irrespective of genotype and the accumulation of *trans*-piceid in all treatments (Additional file 2). As discussed before (chapter 3.2), despite the general induction of *VvSTS* genes in response to an infection, the overall fold changes across the whole set of *VvSTS* genes (Fig. 6b) during a successful defense (in *Rpv3–1/avrRpv3*^*+*^ samples) is significantly up-regulated at 6 hpi compared to susceptible samples (Fig. 6b). This could result in synthesizing a higher level of *trans*-resveratrol that provides the precursors for additional biosynthetic reactions of fungi-toxic oligomeric stilbenes during the *Rpv3–1*-mediated defense response. These genes might therefore not be specific markers for a successful defense while 11 of those 2042 genes were found to be DE specifically in *Rpv3–1/avrRpv3*^*+*^ samples (Fig. 7) when compared to susceptible/*avrRpv3*^*+*^ samples. These 11 genes could only partially be detected using the pairwise statistical approach and they might provide interesting putative marker genes for plants undergoing a successful defense (Table 1). Of these 11 genes, one was functionally characterized as a class III plant peroxidase (VIT_12s0055g01000). Plant peroxidases play a crucial role in many physiological processes and especially in plant defense. Indeed, it was recently demonstrated that peroxidase genes underlay a QTL region that contributes to *Rpv3–3*-mediated resistance to downy mildew [14]. Moreover, it has been suggested that peroxidases are able to catalyze the synthesis of *ε*-viniferin, which was exclusively detected in this study in samples undergoing a successful defense (Fig. 5) [34]. Peroxidases also represent an important class of pathogenesis-related proteins that are able to limit pathogen growth by catalyzing lignification of cell wall components or by producing reactive oxygen species that are involved in hypersensitive response [66, 67]. Two other genes that were found to be differentially expressed in *Rpv3–1/avrRpv3*^*+*^ samples were functionally characterized as aspartyl protease (VIT_04s0008g07150, VIT_04s0008g07250). Even though the role of aspartyl proteases in plants is still hypothetical, some studies have postulated a possible involvement of aspartyl proteases in PCD and autophagocytosis in response to fungal infection [68, 69]. Another DE gene in this group was identified as a GDSL lipase (VIT_10s0003g02120). The physical and molecular functions of GDSL esterases/lipases genes in grapevines are not yet known, but they have been reported to play a role in morphogenesis, plant development, synthesis of secondary metabolites, and plant defense response in other plant species [70, 71]. Moreover a metal-nicotianamine transporter YSL3 (VIT_16s0098g01250) and a chitinase (VIT_05s0062g01320) were found in the group of the 11 DE genes. Chitinases are known pathogen-related proteins playing a role during plant defense even though oomycetes are a less likely target for chitinases, due to the almost absence of chitin in this group of pathogens [72, 73]. In conclusion, the RNA-Seq analysis based on comparative gene expression in an *Rpv3–1* genotype inoculated with virulent and avirulent *P. viticola* isolates has identified genes which might be specifically involved in the early stages of *Rpv3–1-*mediated plant defense and which will be the subject of more detailed examination to determine their putative role in *Rpv3–1* resistance against *P. viticola*.

## Conclusions

Histochemical, transcriptomic and metabolomic analyses of *Rpv3*^+^ and susceptible cultivars inoculated with avirulent and virulent *P. viticola* isolates were performed in this work to investigate mechanism underlying the *Rpv3–1*-mediated resistance response. We demonstrated a strong correlation between the expressions of stilbene biosynthesis related genes, the accumulation of fungi-toxic stilbenes, pathogen growth inhibition and programmed cell death. Our results indicate that pyramiding different *Rpv3* loci can increase the level of resistance to an avirulent downy mildew isolate level but seems not enhance durability of resistance against virulent isolates. Furthermore, several candidate genes potentially involved in *Rpv3*-mediated resistance against *P. viticola* were identified, which will be further studied to unravel the mechanism of resistance.

## Methods

### Plant material, *Plasmopara viticola* isolates and leaf disc infection

Potted grapevines were grown under greenhouse conditions (22 °C/day, 18 °C/night; 50% humidity). *Vitis vinifera* cv. ‘Müller-Thurgau’, ‘Regent’ (*Rpv3–1*) [74], ‘Calardis blanc’ (*Rpv3–1, Rpv3–2*) [15] and ‘Cabernet blanc’ (*Rpv3–1*) (unpublished data) were regenerated from canes obtained from the State Education and Research Center of Viticulture, Horticulture and Rural Development, Neustadt/Weinstr. Germany as described previously [65]. The plant material of this study has been identified and certified by Mr. Neser (Agricultural chamber of Palatinate, Neustadt, Germany) and is deposited in the herbarium of the Julius Kühn-Institut (Bundesforschungsinstitut für Kulturpflanzen, Geilweilerhof, Siebeldingen, Germany). A *P. viticola* isolate that is virulent on *Rpv3* genotypes was originally collected from a commercial ‘Cabernet blanc’ vineyard, whereas an isolate that is avirulent on *Rpv3* genotypes was collected on a susceptible cultivar in Rhineland-Palatinate (Germany) in 2016. According to the classification used previously by Casagrande et al. [16], these isolates were designated *avrRpv3*^*+*^ (avirulent) and *avrRpv3ˉ* (virulent), based on their ability to trigger (or not) cell death on *Rpv3* grapevine genotypes. Isolates were further propagated as described by Malacarne et al. [42]. For all infection experiments, leaf discs (1.5 cm diameter) were excised with a cork borer from the fourth or fifth fully expanded leaves below the shoot apex. Leaf discs were placed upside down on filter paper soaked with 4 ml distilled water (dH_2_O) in a 92 mm diameter petri dish. Freshly harvested sporangia were placed into dH_2_O to release zoospores that were used for inoculation. Four droplets of the zoospore suspension (10 μl each with 40000 sporangia ml^− 1^) or sterile dH_2_O (mock) were placed on the abaxial leaf surface. Droplets were removed with paper 12 h post-inoculation (hpi). Petri dishes were sealed with parafilm and incubated at 22 °C with a photoperiod of 16 h light / 8 h dark until sampling occurred. To reduce the potential contribution of the leaf disc wound surface to changes in gene transcription and metabolite levels, leaf discs were recut (1.3 cm diameter) to remove the outer 2 mm wounded edge, immediately prior to freezing in liquid nitrogen.

### Phenotypic evaluation of resistance to *Plasmopara viticola* isolates

For each treatment, a total of 40 leaf disks were cut from leaves sampled from four individual plant replicates and randomly distributed onto petri dishes prior to inoculation. The development of necrotic lesions was macroscopically scored at 6 days post inoculation (dpi). Additionally, the degree of *P. viticola* infection was quantified by counting the number of sporangia produced per leaf disc at 6 dpi accordingly to Merz et al. [65]. The average of three independent experiments is shown. Averages of each experiment were used for the statistical analysis.

### Histochemical studies

Aniline blue staining was used to monitor *P. viticola* mycelium development according to Hood and Shew [75]. Leaf discs were inoculated with zoospore suspensions as described above. Samples were collected at 24, 48 and 72 hpi and documented with an epifluorescence microscope (ZEISS Axio Scope.A1; Kübler HXP-120C lighting device; Filter set: Zeiss 05; software AxioVision Rel. 4.8). Programmed cell death was studied by trypan blue staining at 24, 28, 32 and 48 hpi as described in Feechan et al. [76]. For a photographic record of leaf disc tissues a ZEISS Axio Lab.A1 microscope with a Zeiss Axiocam MRc camera and Zen blue software were used.

### Determination of stilbene content

Five individual plant replicates of ‘Müller-Thurgau’ and ‘Regent’ were sampled to obtain leaf disks. Each biological replicate was distributed onto a petri dish and leaf disks were inoculated as described above. Two leaf discs per replicate and treatment were pooled together obtaining 10 leaf discs at each time point and treatment. Samples were collected at 0, 6, 24, 48 and 72 hpi and frozen in liquid nitrogen. Extraction was performed as described by Höll et al. [48]. The extracts were separated by HPLC (Knauer Instruments; Smartline autosampler 3800, Smartline pump 1000 and Manager 5000) and stilbene levels were measured with a fluorescence detector (Shimadzu RF-10 AXL). For separation, a Kinetex reversed phase PFP column (2.6 μm, 100 Å, 30 × 2.1 [00A-4477-AN]; Phenomenex) protected by a pre-column was used. Separation was performed with a gradient of solvent A (3% [v/v] acetonitrile; HPLC grade, 96.9% (v/v) water; HPLC grade, 0.1% (v/v) formic acid; HPLC grade) to solvent B (60%[v/v] acetonitrile, 39.9% (v/v) water, 0.1% (v/v) formic acid) to solvent C (80% [v/v] acetonitrile, 19.9% (v/v) water, 0.1% (v/v) formic acid). The gradient conditions were 0 min, 100% solvent A; 25 min, 100% solvent B; 25.5 min, 100% solvent C; 33 min, 100% solvent C; 33.1 min, 100% solvent A; 35 min, 100% solvent A. The column was maintained at RT, and the flow rate was 1.0 ml min-1. Fluorometric detection with a maximum excitation wavelength at 330 nm and emission at 374 nm was used to detect stilbenes as described previously by Pezet et al. [77]. Data acquisition and processing were performed using Clarity Chrom software (Knauer). Calibration curves prepared from commercially available stilbene standards of *trans*-resveratrol, *trans-* piceid, *ε*-viniferin and *trans*- pterostilbene (PhytoLab) were used to calculate stilbene concentrations. The stilbene concentrations were quantified relative to the calibration curve of each standard and expressed as ng g^− 1^ fresh weight (FW) of leaf disc extracted.

### Sampling of leaf discs and total RNA extraction

Five individual plant replicates of ‘Müller-Thurgau’ and ‘Regent’ were sampled to obtain leaf disks. Each biological replicate was distributed onto a petri dish prior to inoculation. At each time point two leaf discs per replicate and treatment were pooled together and collected for RNA extraction, obtaining 10 leaf discs at each time point and treatment. For RNA-Seq analysis an additional experiment with five individual plant replicates was performed to obtain at 6 hpi a second replicate. Leaf discs inoculated with *P. viticola* isolates or treated with H_2_O (mock) were collected at 6, 8, 10, 12, 24 and 48 hpi. Total RNA was isolated with the Spectrum Plant Total RNA purification kit (Sigma Aldrich), following the manufacturer’s instructions and used for qPCR and RNA-Seq analysis. RNA purity (A260/A280 nm) and quantification were measured using a Nanodrop 1000 spectrophotometer (Thermo Fisher Scientific Inc., Wilmington, DE, USA). A qPCR reaction on crude RNA was performed, showing no gDNA contamination.

### Quantitative real time PCR expression analyses

For cDNA synthesis, 350 ng of grapevine total RNA was reverse transcribed using the dART cDNA synthesis kit (Roboklon) as described in Höll et al. [48]. Transcript analysis of genes of interest (GOI) during *P. viticola* infection were determined by qPCR with the SYBR Green method on a Rotor-Gene Q (Qiagen). The PCR reaction mix (15 μl) contained cDNA (1.2 ng), primer (10 μM each), dNTP mix (10 mM each) (Sigma Aldrich), JumpSTART polymerase (2.5 U/μl) (Sigma Aldrich), 0.15 μl from 1:40 dilution SYBR Green in H_2_O (ABsolute™ QPCR SYBR® Green Fluorescein Mix; 1:10 in DMSO; ABgene) and nuclease free water. The thermal cycling conditions used were 95 °C for 6 min followed by 40 cycles of 95 °C for 15 s, 58 °C for 30 s, and 72 °C for 20 s, followed by a melt cycle with 1 °C increments (5 s) from 56 to 96 °C. The primer efficiency was tested with cDNA dilutions of samples. Normalization against the reference genes *VvUbiquitin*, *VvEF1α* and *VvGAPDH* [78] was conducted as described by Pfaffl et al. [79]. The Rotor-Gene Q Series Software Q 2.0.2 (Qiagen) and the Q-Gene software [80] were used for analyzing melt curves and measurement of primer pair efficiency. Gene-specific oligonucleotide sequences are shown in Additional file 6 and representative melting curves are presented in Additional file 7.

### RNA-Seq analysis

#### Preparation of RNA-Seq libraries

For RNA-Seq analysis at 6 hpi, two individual experiments were performed, obtaining two replicates for each treatment with 10 leaf discs pooled from five individual plants for each replicate. Leaf discs of susceptible cultivar ‘Müller-Thurgau’ were inoculated with the avirulent *P. viticola* isolate (*avrRpv3*^+^) or water. Leaf discs of the partially resistant cultivar ‘Regent’ (*Rpv3–1* locus) were inoculated with *avrRpv3*^+^ or *avrRpv3ˉ* isolates or water. From these five experimental conditions, two biological replicates were used for RNA extraction and sequencing library construction resulting in ten samples for RNA-Seq analysis. The quality of the extracted total RNA that was used for library construction was checked with the Agilent 2100 Bioanalyzer (Agilent Technologies) and only RNA samples with an RNA integrity number > 7 were used for library preparation. Libraries for next generation sequencing were prepared using the NEBNext Ultra II Directional RNA Preparation Kit with NEBNext Dual Index Oligo’s for Illumina and the NEBNext Poly A Selection Module (New England Biolabs) according to the manufacturer’s instructions, at the Bioquant, CellNetworks Deep Sequencing Core Facility (Heidelberg, Germany). Single-end sequencing with a length of 75 bp for each read was run on an Illumina NextSeq 500 instrument at the Genomics Core Facility, EMBL (Heidelberg Germany). After sequencing, raw data were transferred to the Quantitative Biology Center (QBiC, https://portal.qbic.uni-tuebingen.de/portal/) at the University of Tübingen using an Aspera client.

#### Quality control, mapping, and differential expression analysis

Initial steps from raw data quality control to mapping and eventually read counting was undertaken by QBiC on the High Performance cluster (HPC) of the University of Tübingen using a fully automated workflow written in Snakemake [81]. The code is accessible here: https://github.com/qbicsoftware/rnaseq. This workflow utilizes the following software packages: FastQC (version 0.11.4) for initial raw data quality control, Cutadapt (version 1.8.3) for filtering reads containing matches to Illumina adapters, Tophat (version 2.2.3.0) for mapping of filtered reads against the reference genome and HTseq-count (version 0.6.1p2) for counting. In the mapping step, reads were aligned to the *Vitis vinifera* reference genome PN40024 [82] downloaded from Ensembl Plants (annotation release 38) in January 2018. Differential expression (DE) analysis was performed using the R packages limma (version 3.32.10) and edgeR (version 3.18.1). First, the raw read count table was filtered for genes that had no expression in any of the samples. Then the remaining counts were normalized by sequencing depth and log_2_-transformed using functions in edgeR to meet the assumptions of linear models. In order to identify differentially expressed genes in *P. viticola* infected versus mock treated samples with respect to susceptibility given by the genetic background (susceptible versus *Rpv3–1*), a linear model was fitted to each gene consisting of a fixed effect for a combined factor of genotype (susceptible versus *Rpv3–1*) and treatment (inoculated with *avrRpv3*^+^ or *avrRpv3ˉ* versus control). This combination of the two main experimental conditions into one factor allowed the extraction of simple contrasts of interest (e.g. susceptible-infected (*avrRpv3*^+^) versus susceptible-control). The same approach allowed the extraction of more complex interaction terms such as [*Rpv3–1_*infected *(avrRpv3*^*+*^*)* versus *Rpv3–1_*control*]* versus [susceptible_infected *(avrRpv3*^*+*^*)* versus susceptible_control] in order to identify which genes respond to infection differently concerning different cultivars. The simple pairwise contrasts as well as more complex interaction terms were extracted from the same statistical model applied to the same dataset. Limma was also used to calculate empirical Bayes moderated *p*-values relative to a minimum required fold-change threshold which were adjusted for multiple testing by controlling the false discovery rate (FDR) ≤0.1% [83].

## Additional files

Additional file 1: *Plasmopara viticola* infection at 24 h post inoculation on leaves of susceptible and *Rpv3–1* cultivars. Germinated sporangia were visualized by UV epifluorescence after aniline blue staining. *P. viticola* spores of the avirulent (*avrRpv3*^*+*^) isolate on the (A) susceptible grapevine cultivar and on (B) *Rpv3–1* cultivar and of the virulent (*avrRpv3ˉ*) *P. viticola* isolate on (C) susceptible grapevine cultivar and (D) *Rpv3–1* cultivar are shown. Images are representative of three biological replicates. Scale bars correspond to 50 μm. (TIFF 369 kb)

Additional file 2: Amount of *trans*-piceid produced in response to *Plasmopara viticola* inoculation. *Trans*-piceid was measured in a susceptible and an *Rpv3–1* cultivar after inoculation with *P. viticola* isolates (*avrRpv3*^*+*^ or *avrRpv3¯*) or treatment with water (H_2_O). Samples were collected 0, 6, 24, 48 and 72 hpi. Each bar represents the mean of four biological replicates. Bars represents the average of one experiment with four biological replicates and two independent measurements. Error bars show standard deviation. ANOVA was used to determine the effects of cultivar and treatment (the two isolates) on the stilbene amount and then means were compared by Tukey’s HSD test. Statistical analysis is related to significance of all samples at the same time point, different letters (a, b, c) are significantly different (*P* < 0.05). (TIFF 105 kb)

Additional file 3: Pairwise comparison analysis using LIMMA of *Vitis vinifera* gene expression in response to *Plasmopara viticola* infection. Blue font indicates significant up-regulation, while red font highlights significant down-regulation (adjusted *P* ≤ 0.1). Gray font denotes genes with fold changes that were not significant (adjusted *P* > 0.1). FC, fold change. (XLSX 460 kb)

Additional file 4: Comparison of RNA-Seq and real-time qPCR analyses. Scatterplot of the correlation between normalized counts (*P. viticola* vs mock) of four expressed genes (*VvPR10.1*, *VvPR5*, *VvROMT* and *VvSTS1*) as assessed by RNA-Seq analysis and the relative expression levels (fold-change relative to the expression in control plants and normalized against housekeeping genes) as assessed by qPCR. (A) *Rpv3–1* (*avrRpv3*^*+*^ vs mock), (B) susceptible (*avrRpv3*^*+*^ vs mock) and (C) *Rpv3–1* (*avrRpv3¯* vs mock). A linear trend is shown. (TIFF 93 kb)

Additional file 5: List of 2042 differentially expressed genes identified using interaction term analysis and displayed in Venn diagram (Fig. 7). xInteraction term analyses to identify DEG characteristic for a successful defence by comparing the pairwise contrasts of *Rpv3–1* samples with susceptible samples. (adjusted P ≤ 0.1). FC, fold change. (XLSX 305 kb)

Additional file 6: Sequence of the oligonucleotides used for qPCR analysis. (XLSX 9 kb)

Additional file 7: Melting curves of oligonucleotides used for qPCR analysis. Description of data: Pictures show a representative melting curve of a cDNA template (red) and the negative control (light blue) of (A) *VvEF1α*, (B) *VvGAPDH*, (C) *VvUbiquitin*, (D) *VvSTS25/27/29*, (E) *VvROMT*, (F) *VvPR10.1*, (G) *VvPR5* and (H) *VvSTS1*. x axis shows the temperature (°C) and y axis the change in fluorescence level with respect to temperature increase (dF/dT). (TIF 12742 kb)

## Data Availability

Data from this project was also deposited in Gene Expression Omnibus (GEO) of the National Center for Biotechnology Information (http://www.ncbi.nlm.nih.gov/geo/) with the following accession number: GSE128865.

## Abbreviations

*avrRpv3ˉ*: Virulent *Plasmopara viticola* isolate
*avrRpv3*^*+*^: Avirulent *Plasmopara viticola* isolate
DE: Differentially expressed
dpi: Days post inoculation
ETI: Effector-triggered immunity
hpi: Hours post inoculation
HR: Hypersensitive response
PAMPs: Pathogen associated molecular patterns
PCD: Programmed cell death
QTL: Quantitative trait loci
ROS: Reactive oxygen species
*Rpv*: Resistance to *Plasmopara viticola*
