# Supplementary figures and images for: Rpv3–1 mediated resistance to grapevine downy mildew is associated with specific host transcriptional responses and the accumulation of stilbenes
Source: BMC Plant Biol. 2019 Aug 6;19:343. doi: 10.1186/s12870-019-1935-3 (PMC6685164; doi:10.1186/s12870-019-1935-3)

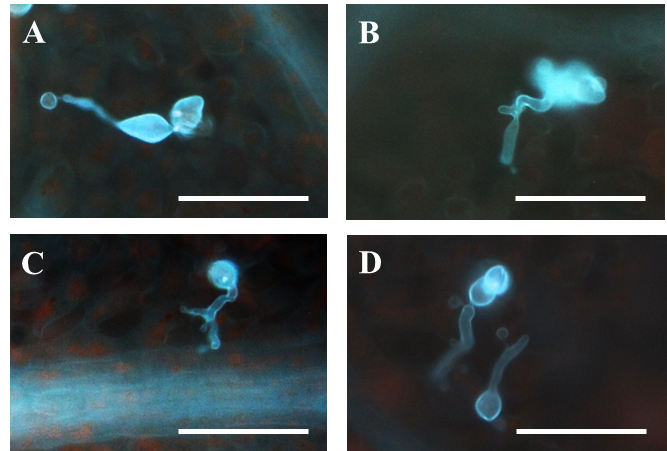

Supplement: Supplementary file 1 — Plasmopara viticola infection at 24 h post inoculation on leaves of susceptible and Rpv3–1 cultivars. Germinated sporangia were visualized by UV epifluorescence after aniline blue staining. P. viticola spores of the avirulent (avrRpv3+) isolate on the (A) susceptible grapevine cultivar and on (B) Rpv3–1 cultivar and of the virulent (avrRpv3ˉ) P. viticola isolate on (C) susceptible grapevine cultivar and (D) Rpv3–1 cultivar are shown. Images are representative of three biological replicates. Scale bars correspond to 50 μm. (TIFF 369 kb) [file 12870_2019_1935_MOESM1_ESM.tiff]

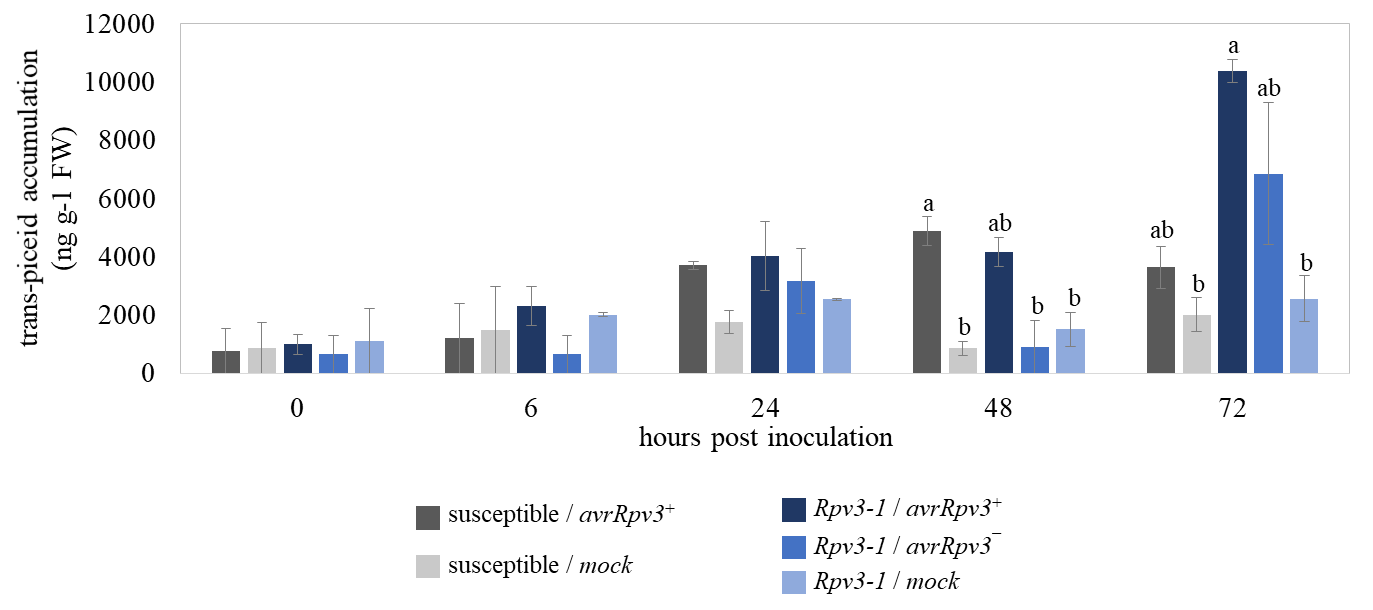

Supplement: Supplementary file 2 — Amount of trans-piceid produced in response to Plasmopara viticola inoculation. Trans-piceid was measured in a susceptible and an Rpv3–1 cultivar after inoculation with P. viticola isolates (avrRpv3+ or avrRpv3¯) or treatment with water (H2O). Samples were collected 0, 6, 24, 48 and 72 hpi. Each bar represents the mean of four biological replicates. Bars represents the average of one experiment with four biological replicates and two independent measurements. Error bars show standard deviation. ANOVA was used to determine the effects of cultivar and treatment (the two isolates) on the stilbene amount and then means were compared by Tukey’s HSD test. Statistical analysis is related to significance of all samples at the same time point, different letters (a, b, c) are significantly different (P < 0.05). (TIFF 105 kb) [file 12870_2019_1935_MOESM2_ESM.tiff]

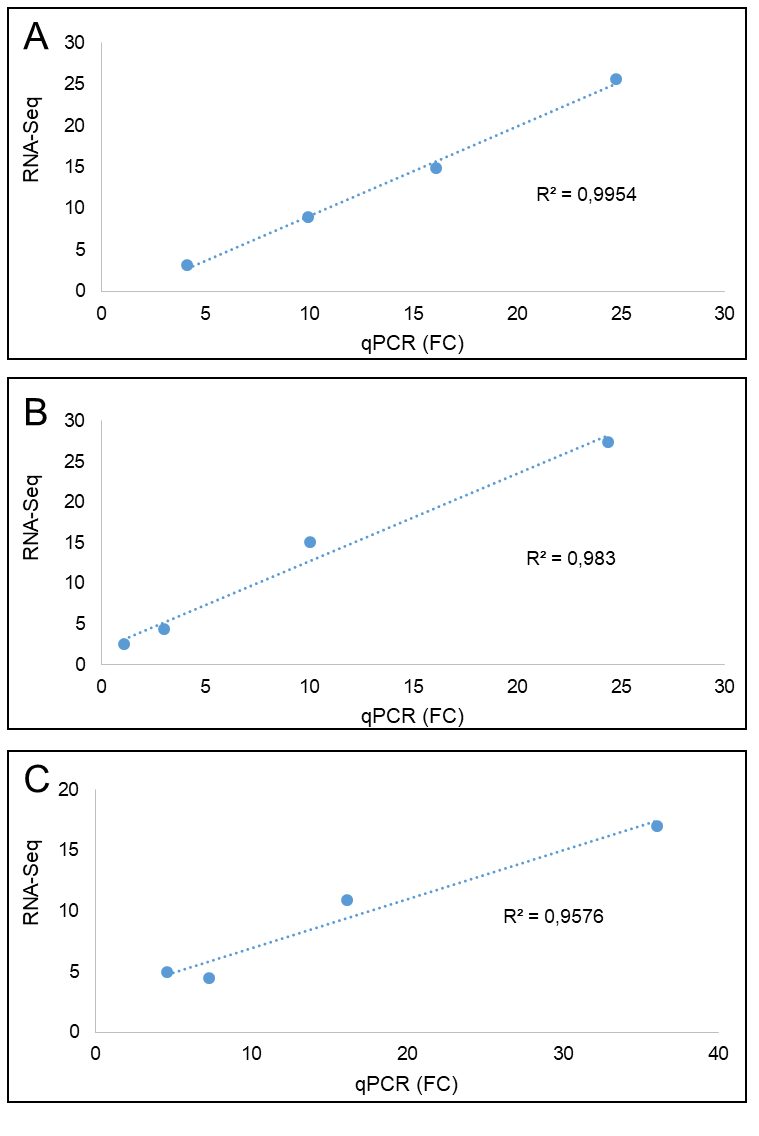

Supplement: Supplementary file 4 — Comparison of RNA-Seq and real-time qPCR analyses. Scatterplot of the correlation between normalized counts (P. viticola vs mock) of four expressed genes (VvPR10.1, VvPR5, VvROMT and VvSTS1) as assessed by RNA-Seq analysis and the relative expression levels (fold-change relative to the expression in control plants and normalized against housekeeping genes) as assessed by qPCR. (A) Rpv3–1 (avrRpv3+ vs mock), (B) susceptible (avrRpv3+ vs mock) and (C) Rpv3–1 (avrRpv3¯ vs mock). A linear trend is shown. (TIFF 93 kb) [file 12870_2019_1935_MOESM4_ESM.tiff]

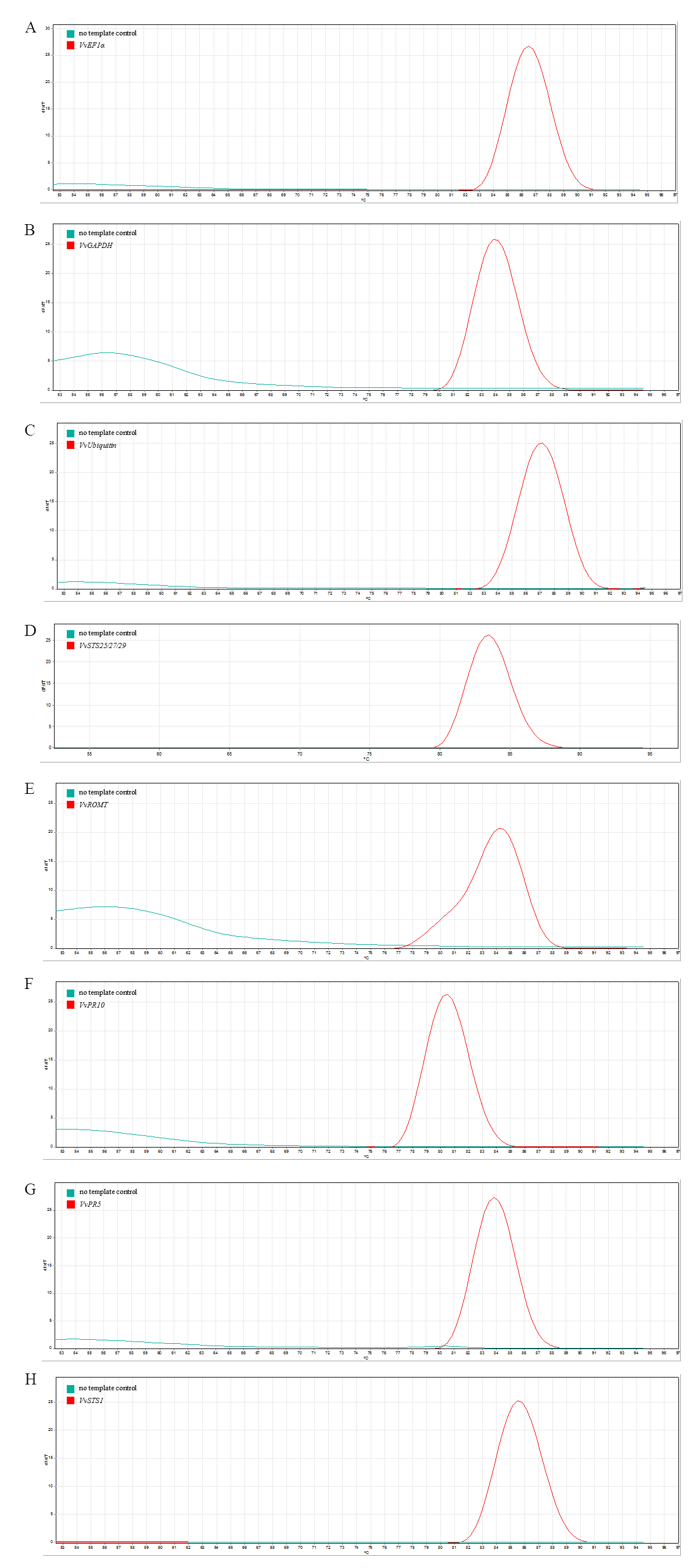

Supplement: Supplementary file 7 — Melting curves of oligonucleotides used for qPCR analysis. Description of data: Pictures show a representative melting curve of a cDNA template (red) and the negative control (light blue) of (A) VvEF1α, (B) VvGAPDH, (C) VvUbiquitin, (D) VvSTS25/27/29, (E) VvROMT, (F) VvPR10.1, (G) VvPR5 and (H) VvSTS1. x axis shows the temperature (°C) and y axis the change in fluorescence level with respect to temperature increase (dF/dT). (TIF 12742 kb) [file 12870_2019_1935_MOESM7_ESM.tif]
